# Potential Roles of Exosomes in Parkinson’s Disease: From Pathogenesis, Diagnosis, and Treatment to Prognosis

**DOI:** 10.3389/fcell.2020.00086

**Published:** 2020-02-21

**Authors:** Haiyang Yu, Tong Sun, Jing An, Lulu Wen, Fei Liu, Zhongqi Bu, Yueran Cui, Juan Feng

**Affiliations:** ^1^Department of Neurology, Shengjing Hospital of China Medical University, Shenyang, China; ^2^Department of Neonatology, Shengjing Hospital of China Medical University, Shenyang, China

**Keywords:** exosomes, Parkinson’s disease, neurodegeneration, neuroinflammation, pathogenesis, diagnosis, treatment, prognosis

## Abstract

Parkinson’s disease (PD) is the second most prevalent neurodegenerative disease in the world, after Alzheimer’s disease (AD), affecting approximately 1% of people over 65 years of age. Exosomes were once considered to be cellular waste and functionless. However, our understanding about exosome function has increased, and exosomes have been found to carry specific proteins, lipids, functional messenger RNAs (mRNAs), high amounts of non-coding RNAs (including microRNAs, lncRNAs, and circRNAs) and other bioactive substances. Exosomes have been shown to be involved in many physiological processes *in vivo*, including intercellular communication, cell migration, angiogenesis, and anti-tumor immunity. Moreover, exosomes may be pivotal in the occurrence and progression of various diseases. Therefore, exosomes have several diverse potential applications due to their unique structure and function. For instance, exosomes may be used as biological markers for the diagnosis and prognosis of various diseases, or as a natural carrier of drugs for clinical treatment. Here, we review the potential roles of exosomes in the pathogenesis, diagnosis, treatment, and prognosis of PD.

## Introduction

Parkinson’s disease (PD) is the second most common neurodegenerative disease in the world, after Alzheimer’s disease (AD) (Kalia and Lang, 2015). PD affects 0.3% of the whole population, and the percentage rises to 1% of the population above 65 years of age (De Lau and Breteler, 2006; Ascherio and Schwarzschild, 2016). The clinical symptoms of PD patients include resting tremor, muscular rigidity, bradykinesia, and postural instability. Prior to the eventual appearance of motor symptoms, non-motor symptoms can be observed in PD patients, including hyposmia, constipation, urinary dysfunction, depression, anxiety, and rapid eye movement sleep behavior disorder (RBD) (Schapira et al., 2017). Most cases of PD are idiopathic, although 10–15% of cases are genetic (Deng et al., 2018). Thus far, 23 genes related to PD (PARK genes) have been identified. Mutations in SNCA, LRRK2, and VSP32 cause autosomal dominant inheritance of PD, whereas mutations in PRKN, PINK1, and DJ-1 demonstrate autosomal recessive inheritance. Moreover, a mutation in glucocerebrosidase 1 (GBA1) which causes Gaucher disease has been identified as a potential genetic risk factor for PD (Nichols et al., 2009), and both Gaucher disease patients and obligate carriers are predisposed to PD. In addition to genetic factors, environmental factors are also crucial in PD pathogenesis. For example, personal exposure to noxious chemicals such as 1-methyl-4-phenyl-1,2,3,6-tetrahydropyridine (MPTP) (Langston et al., 1983), rotenone (Betarbet et al., 2000), and paraquat (Elbaz et al., 2009; Tanner et al., 2011) can trigger PD symptoms. The risks for developing PD associated with heavy mental exposure were also investigated, but the correlation remained unclear. Notably, some studies have reported a reduced risk of developing PD among smokers (Hernán et al., 2001) and coffee drinkers (Ross et al., 2000; Palacios et al., 2012).

The hallmarks of PD pathology are lesions in the substantia nigra pars compacta (SNpc) and striatum, including the presence of cytoplasmic inclusion bodies known as Lewy bodies (LBs), and progressive loss of dopaminergic neurons (Dickson, 2012). LBs are mainly composed of filamentous α-synuclein (α-syn), a small protein of indefinite function that is ubiquitously expressed in the brain. In PD patients, α-syn becomes abnormally phosphorylated and aggregated (Spillantini et al., 1998). PD movement disorders become more and more severe as the disease progresses due to the continuing death of dopaminergic neurons in SNpc, even though other brain regions are also affected. Although α-syn misfolding and aggregation are central to the development of the disease, several other mechanisms are implicated in PD pathogenesis (Goedert et al., 2013). Indeed, mitochondrial dysfunction (Bose and Beal, 2016), neuroinflammation (Hirsch and Hunot, 2009), and abnormal protein clearance systems, including the ubiquitin-proteasome system (UPS) (McKinnon and Tabrizi, 2014) and the autophagy-lysosomal pathway (ALP) (Tanji et al., 2011), all play a role in PD pathogenesis. Although all of these processes are known to participate in the onset and progression of PD, the relationship between these pathways remains unknown.

Extracellular vesicles (EVs) were first described in plasma by Wolf some 50 years ago as “platelet dust” (Wolf, 1967). There are three main types of EVs and these are distinguished by their size and their different mechanisms of biogenesis and release. Microvesicles (MVs) and apoptotic bodies are 50–500 nm in diameter, and both are secreted directly from the cytoplasmic membrane by living or dying cells (Colombo et al., 2014). Exosomes, the smallest EVs, are 50–150 nm in diameter and are secreted from the cytoplasmic membrane into the extracellular environment via fusion with late endosomes or multivesicular bodies (MVBs). This type of vesicle was first identified in the rat (Harding et al., 1983) and sheep (Pan et al., 1985) reticulocytes and subsequently labeled as “exosomes” by Johnstone et al. (1987) and Johnstone (2005). Exosomes can be released by a wide range of cells, including neurons, blood cells, epithelial cells, immune cells, and cancer cells. Exosomes have been isolated from a variety of biological fluids, including cerebrospinal fluid (CSF), plasma, serum, saliva, urine, semen, breast milk, amniotic fluid, and ascites fluid. Besides, exosomes have been isolated *in vitro* from cell culture supernatants (Théry et al., 2006). This review aimed to examine the potential roles of exosomes in the pathogenesis, diagnosis, treatment, and prognosis of PD. Since circulating exosomes cannot definitively be distinguished from MVs using currently available purification methods, we refer to EVs rather than exosomes when referring to biomarker studies in this review.

## Biogenesis and Release of Exosomes

### Exosome Biogenesis: Formation of Intraluminal Vesicles (ILVs) in MVBs and Cargo Sorting

**FIGURE 1:**
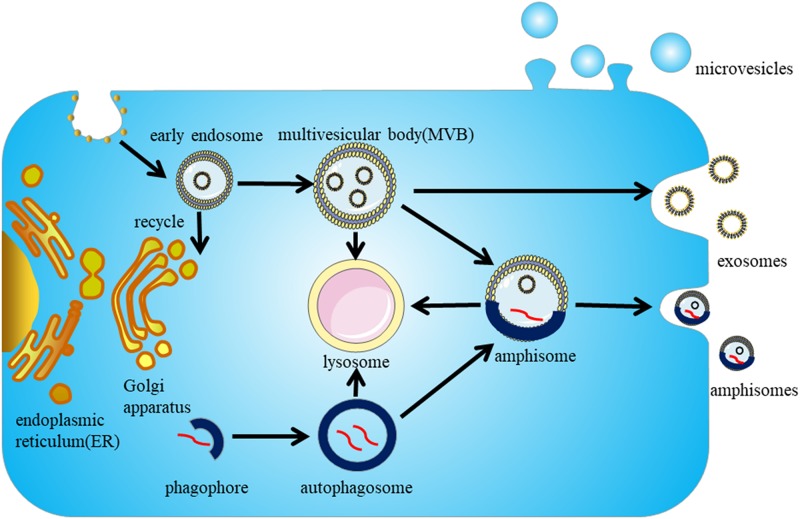
Biogenesis and secretion of exosomes. Exosome biogenesis begins in the endosome pathway. The cytoplasmic membrane invaginates to form early endosomes, parts of early endosomes are recycled by the Golgi apparatus, but the majority of them mature into late endosomes (also called MVBs). The intraluminal vesicles (ILVs) in MVBs either secrete to the extracellular environment to form exosomes or are targeted to lysosomes for degradation. The autophagy pathway is proposed to interact with the endosome pathway, and the autophagosomes and MVBs can fuse to form amphisomes, which can be degraded by lysosomes or secreted extracellularly.

Exosome biogenesis begins with the endosome system. The cytoplasmic membrane invaginates to form early endosomes, and while some are recycled by the Golgi apparatus, the majority of early endosomes develop into late endosomes (also called MVBs). ILVs are formed during the inward budding of the endosomal membrane into the lumen of MVBs (Klumperman and Raposo, 2014) (Figure 1). The whole process can be divided into ESCRT-dependent and ESCRT-independent mechanisms; the molecules involved in the biogenesis and release of exosomes are summarized in Table 1.

**TABLE 1:** Molecules involved in the biogenesis and secretion of exosomes.

**Molecules**	**Cell lines**	**Functions**	**References**
**ESCRT-dependent pathway**			
**ESCRT-0**			
HRS	HeLa cells, HEK293 cells, Head and neck squamous cell carcinoma, Mouse dendritic cells	Depletion of HRS can reduce the exosome secretion	Tamai et al., 2010; Gross et al., 2012; Colombo et al., 2013; Hoshino et al., 2013
STAM	HeLa cells	Depletion of STAM1 can reduce exosome secretion	Colombo et al., 2013
**ESCRT-I**			
TSG101	HeLa cells, MCF-7 cells	Knockdown of TSG101 results in reduced exosome secretion	Baietti et al., 2012; Colombo et al., 2013
**ESCRT-II**	HeLa cells	unclear	
**ESCRT-III**			
CHMP4	MCF-7 cells	Knockdown of the ESCRT-III component CHMP4 can decrease exosome secretion	Baietti et al., 2012
**Accessory proteins**			
ALIX	HeLa cells	Silencing of ALIX does not affect the number of exosomes secreted, but changes the protein composition of exosomes, increasing the number of MCH class II molecules in cells and exosomes	Colombo et al., 2013
	Mouse dendritic cells	Silencing of ALIX reduced exosomes containing MHC class II, CD63, and CD81 in approximately half of the cells	Tamai et al., 2010
	MCF-7 cells	The syndecan–syntenin–ALIX interaction was necessary for biogenesis of exosomes	Friand et al., 2015
VPS4	HeLa cells	Inhibition of VPS4B led to increased exosome secretion	Colombo et al., 2013
	MCF-7 cells	Simultaneously inhibition of VSP4A and VSP4B reduced exosome secretion	Baietti et al., 2012
**ESCRT-independent pathway**			
**Lipids**			
Ceramide	Oligodendroglia cells	Inhibition of sphingomyelinase can impair the synthesis of ceramide, thus disrupting the secretion of exosomes	Trajkovic et al., 2008
Phospholipase D2(PLD2)	RBL-2H3 cells	Inhibition of PLD2 can impair the synthesis of PA, thus disrupting the secretion of exosomes	Laulagnier et al., 2004
Cholesterol	Oligodendroglia cells	Accumulation of cholesterol in MVBs can induce secretion of exosomes expressing CD63, ALIX, and Flotillin-2, from oligodendroglia cells	Strauss et al., 2010
**Tetraspanin family**			
CD9	HEK293 cells	CD9 has been shown to increase exosome secretion	Chairoungdua et al., 2010
	Dendritic cells	In the CD9 knockout mouse, secretion of exosomes expressing flotillin-1 is decreased from bone marrow dendritic cells.	Chairoungdua et al., 2010
CD63	Melanoma cells	CD63 has been shown to sort the melanosomal protein PMEL into ILVs	Van Niel et al., 2011
CD81	Lymphoblasts	CD81 ligands are transported to exosomes for secretion	Perez-Hernandez et al., 2013
CD82	HEK293 cells	CD82 has been shown to increase exosome secretion	Chairoungdua et al., 2010
TSPAN8	Rat adenocarcinoma cells	Overexpression of TSPAN8 changed the mRNA and protein constituents in exosomes, without affecting the amount of exosome secretion	Nazarenko et al., 2010
**Chaperone**			
Hsc70	Reticulocytes	Hsc70 was shown to recruit transferrin receptor (TFR) in mature reticulocytes	Géminard et al., 2004
**Rab GTPase family**			
Rab2B, Rab5A, Rab9A	HeLa cells	knockdown of Rab2B, Rab5A, and Rab9A can reduce the secretion of exosomes	Ostrowski et al., 2010
Rab7	HeLa cells	Knockdown of Rab7 dis not influence exosome secretion	Ostrowski et al., 2010
	MCF-7 cells	Rab7 is involved in the release of exosomes containing syntenin and ALIX	Baietti et al., 2012
Rab11	HeLa cells	Knockdown of Rab11A did not influence exosome secretion	Ostrowski et al., 2010
	K562 cells	Rab11 was linked to the exosome secretion involving TFR and Hsc70	Savina et al., 2002
	RPE1 cells	Rab11 is involved in the release of exosomes containing anthrax toxin	Abrami et al., 2013
Rab27A or Rab27B	HeLa cells	Depletion of Rab27A or Rab27B, located in late endosomes and lysosome-related organelles, strikingly decreased the amount of exosome secretion	Ostrowski et al., 2010
Rab35	Oligodendroglia cells, Oli-neu cells	Knockdown of Rab35 interferes with the processing of PLP-expressing exosomes in oli-neu cells and primary oligodendrocytes	Hsu et al., 2010; Frühbeis et al., 2013
	RPE1 cells	Rab35 is involved in the release of exosomes containing anthrax toxin	Abrami et al., 2013
**SNAREs family**			
VAMP7	K562 erythroleukemia cells	Exosome secretion depends on v-SNARE protein VAMP7	Fader et al., 2009
	MDCK cells	Inhibition of VAMP7 disrupted release of lysosomes but not secretion of exosomes	Proux-Gillardeaux et al., 2007
YKT6	HEK293 cells	v-SNARE protein YKT6 is essential for release of exosomes containing the WNT3A morphogen	Gross et al., 2012

The Endosomal Sorting Complex Required for Transport (ESCRT) mechanism was first identified in the 2000s (Katzmann et al., 2001). The ESCRT system is composed of four complexes ESCRT-0, -I, -II, -III, and their associated proteins. ESCRT-0 proteins HRS and STAM are involved in cargo sorting of ubiquitinated proteins (Hanson and Cashikar, 2012; Henne et al., 2013). ESCRT-I proteins TSG101 and ESCRT-II are responsible for bud formation. ESCRT-III induces vesicle scission.

In the ESCRT system, dissociation and recycling of vesicles is driven by the accessory proteins ALIX and VPS4. ALIX was recently found to interact with syntenin, which acts as a cytoplasmic adaptor of heparan sulfate proteoglycan receptors. VPS4 is an ATPase associated with various cellular activities (AAA + ATPase) and is involved in the final step of ILV formation.

In addition to ESCRT-dependent mechanisms, some studies have shown that exosome biogenesis can occur in the absence of ESCRT proteins. Lipids, tetraspanins, and heat shock proteins are all involved in ESCRT-independent mechanisms (Stuffers et al., 2009). After inhibiting the expression of ESCRT proteins, exosomes expressing proteolipid protein (PLP) are normally released by oligodendroglia cells. Sphingomyelinase, an enzyme involved in producing ceramide from sphingomyelin, plays a role in exosome biogenesis and secretion in oligodendroglia cells (Trajkovic et al., 2008). These findings are consistent with the high levels of ceramide in exosomes. Ceramides are proposed to facilitate inward budding of MVBs to form ILVs. Phospholipase D2 (PLD2), an enzyme involved in synthesizing the signal molecule phosphatidic acid (PA) from phospholipids, also influences exosome biogenesis (Laulagnier et al., 2004). As observed with ceramide, PA can also induce inward curvature of the MVB membrane to form ILVs. Lipids such as cholesterol are also enriched in exosomes (Strauss et al., 2010).

In addition to lipids, the Tetraspanin family of four transmembrane domain proteins, which are enriched in exosomes, have also been proposed to participate in sorting cargoes for exosome secretion. Finally, the chaperone Hsc70 also plays a role in cargo sorting for exosomes. Hsc70 mediates the association between cytosolic constituents and exosomal membrane proteins (Géminard et al., 2004). Recently, Hsc70 was shown to bind cytosolic proteins containing a KFERQ-motif, and to induce their selective transport to ILVs (Sahu et al., 2011).

Although the biogenesis of exosomes is divided into ESCRT-dependent and ESCRT-independent pathways, the two pathways cannot be totally separated. Moreover, multiple mechanisms may simultaneously occur in a single MVB, and different populations of exosomes may depend on different pathways.

### Exosome Secretion: Transportation of MVBs to the Cytoplasmic Membrane and Subsequent Fusion of MVBs With the Cytoplasmic Membrane

After ILVs have formed in MVBs, the MVBs can either be transported to lysosomes for degradation or directed to the cytoplasmic membrane for exosome secretion. Transportation of MVBs to the cytoplasmic membrane requires actin and microtubule cytoskeletons and associated molecular motors, such as kinesins and myosins (Villarroya-Beltri et al., 2014). Silencing or overexpressing cortactin, an actin-binding protein, was confirmed to reduce or increase the exosome secretion, respectively (Sinha et al., 2016).

Members of the Rab GTPase family, the largest family of small GTPase proteins, play vital roles in several critical MVB transportation processes, including vesicles membrane budding, trafficking of vesicles with tubulin and actin, docking of vesicles to targeted compartments, and fusion of vesicles with the cytoplasmic membrane (Stenmark, 2009).

Upon transportation of MVBs to the cytoplasmic membrane, MVBs fuse with the cytoplasmic membrane. During this process, numerous interactions between proteins and lipids can reduce the energy barrier and facilitate the fusion. Soluble *N*-ethylmaleimide-sensitive factor attachment proteins receptors (SNAREs) complexes are intimately involved in fusion with intracellular membranes (Zylbersztejn and Galli, 2011). The SNAREs family is comprised of vesicle-associated SNAREs (v-SNAREs) and target membrane-associated SNAREs (t-SNAREs). Generally, during the membrane fusion process, v-SNAREs interact with t-SNAREs to form a trans-SNARE complex (Wang et al., 2017). SNARE proteins including vesicle-associated membrane protein 7 (VAMP7), vesicle-associated membrane protein 8 (VAMP8), and SNAP-23 are involved in exocytosis of lysosomes regulated by Ca^2+^ (Puri and Roche, 2008). However, whether SNAREs induce the fusion of MVBs with the cytoplasmic membrane is controversial.

Cell type and cellular homeostasis also have an essential effect on the pathways of exosome secretion. MVBs are targeted to lysosomes for degradation or to the cytoplasmic membrane for secretion depending on cellular homeostasis. Under stress conditions, such as hypoxia (King et al., 2012), irradiation (Lehmann et al., 2008), and ER stress (Kanemoto et al., 2016), the number of intracellular MVBs increase and more exosomes are released. Thus, exosome secretion may be an alternative route for stressful cells to clear waste products. The secreted exosomes may be degraded by phagocytes, or may communicate with neighboring cells to induce pathological conditions. Similarly, an association between exosome secretion and autophagy has been proposed. Autophagy is a degradative pathway for the elimination of cellular waste, damaged organelles, and aggregated proteins, and functions to maintain cellular homeostasis. The cargoes to be excreted are first packaged into autophagosomes, and the autophagosomes are then either directly targeted to lysosomes for degradation or fused with MVBs to form amphisomes. The amphisomes can be degraded in lysosomes or secreted extracellularly (Boya et al., 2013). The two pathways do somewhat overlap. However, the induction of autophagy by starvation can decrease exosome secretion. Moreover, inhibition of autophagic degradation induced by inhibiting the fusion of lysosomes with both autophagosomes and MVBs could enhance the secretion of exosomes (Fader et al., 2008).

## Role of Exosomes in the Pathogenesis of PD

**FIGURE 2:**
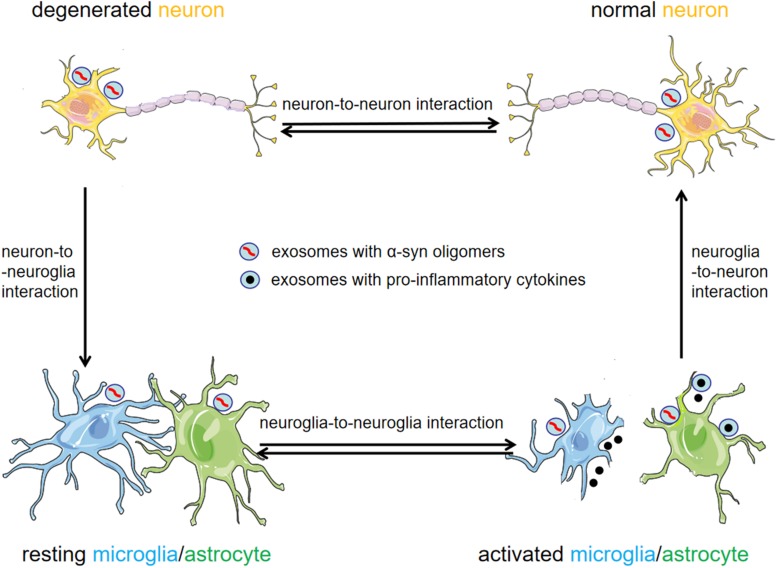
Exosomes as mediators for cell-to-cell communication in the pathogenesis of PD. Exosomal α-syn is readily transmitted between neurons and neuroglia cells. Exosomes provide an environment for α-syn aggregation, and can potentially promote the propagation of α-syn oligomers in the CNS. Activated neuroglia cells increase the release of exosomes and pro-inflammatory cytokines, thus exacerbating neuroinflammation and the progression of PD.

Parkinson’s disease is characterized by the histopathological formation of LBs which predominantly contain misfolded α-syn. The conspicuous motor symptoms occur because of the progressive loss of dopaminergic neurons in the SNpc and striatum. However, preceding motor symptoms, patients can display some non-motor symptoms including hyposmia, gastrointestinal dysfunction, and sleep disorders (Khoo et al., 2013). Although LBs are initially found in the periphery, they gradually spread to the brain stem, and ultimately to the cerebral cortex. Following these observations, a hypothesis has been proposed that PD may begin in the enteric nervous system or olfactory bulbs and then spread to other brain regions during disease progression (Braak et al., 2003b; Del Tredici and Braak, 2016). Currently, PD progression is unexplained, but α-syn has been thought to propagate in a prion-like process (Olanow and Brundin, 2013). Moreover, exosomes are proposed to play an important role in the progression of PD (Figure 2).

### α-syn Aggregation and Propagation Mediated by Exosomes

**FIGURE 3:**
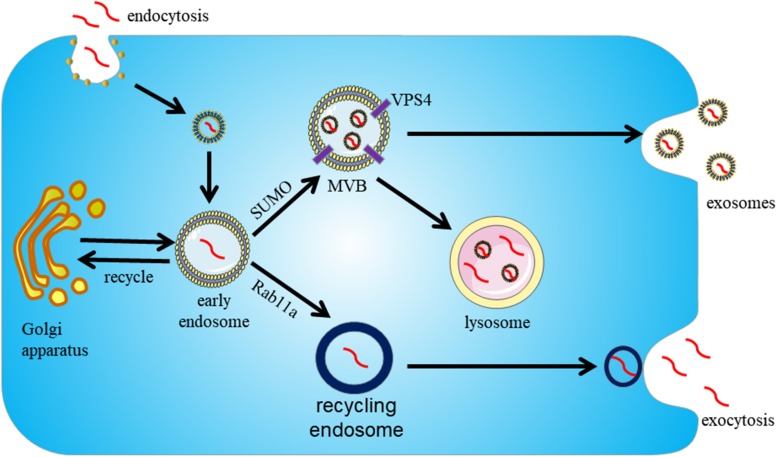
Proposed pathway for sorting α-syn into exosomes. Exosomes participate in the secretion of α-syn in an endosome-dependent mechanism. First, α-syn is packaged into early endosomes. Next, α-syn containing exosomes can be secreted by two pathways. With the assistance of VPS4 and SUMO, it can be secreted as exosomal cargoes upon fusion of MVBs with the cytoplasmic membrane. Alternatively, α-syn containing exosomes can be sorted into recycling endosomes and be exocytosed as secretory granules in a Rab11a-dependent way.

Several experiments showed that α-syn is directly released into the extracellular environment or packaged into exosomes via the endosome pathway (Lee et al., 2005). However, the underlying mechanisms by which α-syn is sorted into exosomes remains unclear. In addition, α-syn reportedly interacts with synaptic vesicles to facilitate SNAREs assembly and promote neurotransmitter release (Alvarez-Erviti et al., 2010). The synaptic vesicles containing α-syn can be sorted into early endosomes by the Golgi apparatus or clathrin-mediated endocytosis (Alvarez-Erviti et al., 2011b). Next, with the assistance of VPS4 and SUMO (Small Ubiquitin-like Modifier) proteins, α-syn containing endosomes transition into MVBs and fuse with the plasma membrane for secretion as exosomal cargoes (Cabin et al., 2002). Alternatively, α-syn containing endosomes can be sorted into recycling endosomes and be exocytosed as secretory granules in a Rab11a-dependent manner (Ben Gedalya et al., 2009). In all processes, the secretion of exosomal α-syn from cells is regulated by the intracellular calcium concentration (Emmanouilidou et al., 2010) (Figure 3).

Although the levels of α-syn contained in exosomes are low, recent studies suggest that exosomes provide an ideal environment for α-syn to aggregate, and potentially promote the propagation of PD pathology (Grey et al., 2015). It is widely accepted that oligomeric α-syn is the toxic form of α-syn, responsible for neuronal death. Danzer et al. (2012) identified the presence of oligomeric α-syn in exosomes and demonstrated that α-syn in exosomes is more easily taken up by recipient cells than free α-syn (Danzer et al., 2012). Stuendl et al. (2016) reported that exosomes from the CSF of patients with PD and dementia with Lewy bodies (DLB) could induce the formation of α-syn oligomers (Stuendl et al., 2016). Together, these findings prove that exosomal α-syn is intimately involved in the transmission of α-syn oligomers between cells.

Exosomes may function as intercellular carriers allowing α-syn to propagate in PD patients. Li et al. (2008) found LBs formation in transplanted neurons within recipients with PD, suggesting disease propagation from host-to-graft (Li et al., 2008). In the long-term embryonic nigral transplants in PD, Kordower et al. (2008) observed LB-like pathology (Kordower et al., 2008). Moreover, Recasens et al. (2014) extracted LBs from the brains of PD patients and transplanted them into mice and monkeys, eventually triggering α-syn aggregation and neurodegeneration (Recasens et al., 2014). In *in vitro* experiments, α-syn in exosomes were isolated from the conditioned medium of SH-SY5Y cells following overexpression of wild-type α-syn. Subsequently, Alvarez-Erviti et al. (2011b) demonstrated that exosomes isolated from SH-SY5Y cells which overexpress α-syn could transfer α-syn to normal SH-SY5Y cells (Alvarez-Erviti et al., 2011b). Finally, Emmanouilidou et al. (2010) were able to show that α-syn containing exosomes can promote the cell death of recipient neuronal cells, providing support for the hypothesis that α-syn propagation between neurons facilitates PD progression (Emmanouilidou et al., 2010).

Many different pathways are involved in the secretion of α-syn by exosomes. One of the characteristics of PD pathology is the failure of protein clearance by the UPS and the ALP. If either of the two pathways is compromised, intracellular α-syn aggregation occurs (Alvarez-Erviti et al., 2011b). Inhibition of ALP has been found to increase exosomal α-syn release, while concomitantly reducing α-syn aggregation in the cell (Lee et al., 2013). Although inhibition of ALP can protect cells by the reduction of intracellular α-syn levels and an increase in exosomal α-syn secretion, it can also promote the intercellular transfer of α-syn by exosomes and lead to the propagation of PD pathology (Poehler et al., 2014).

### Neuroinflammation Mediated by Exosomes

In addition to neuron-to-neuron communication, exosomes can also communicate between neurons and neuroglia cells. It has been reported that α-syn secreted from neurons can be phagocytosed by microglial cells (Alvarez-Erviti et al., 2011a) and astrocytes (Lee et al., 2010) in order to eliminate the toxic α-syn. However, the overload of α-syn in neuroglia cells can trigger a neuroinflammatory response. Chang et al. (2013) reported that α-syn could induce increased exosome release by BV-2 microglial cells, and this can cause increased cell apoptosis when co-cultured with cortical neurons (Chang et al., 2013). In addition, microglial cells and monocytes isolated from young (but not old) mice showed increased phagocytosis of exosomal α-syn oligomers and decreased secretion of the pro-inflammatory cytokines TNF-α (Bliederhaeuser et al., 2016). These studies suggest that microglia cells in the aged brain can exacerbate neurodegeneration by being unable to eliminate α-syn oligomers or by accelerating the secretion of exosomal α-syn oligomers. All these findings suggest that exosomes secreted by activated microglia cells may be vital factors in the neurodegeneration and progression of PD.

Inflammation is a critical process in PD progression (Panaro and Cianciulli, 2012). A proper inflammatory response is essential for tissue repair, but an excessive and delayed inflammatory response may lead to a malignant cycle of neuroinflammation and propagation of the disease (Gao and Hong, 2008). Exosomes may participate in different stages of the inflammatory process, including the activation stage, through neuron-to-neuroglia communication, and the exacerbation stage, through neuroglia-to-neuroglia communication (Figure 2). Thus far, the exact role of exosomes in neuroinflammation has not been completely elucidated, and more research is essential.

### MicroRNAs in Exosomes Involved in PD Pathogenesis

MicroRNAs (miRNAs) are notoriously involved in PD pathogenesis, and can be packaged in exosomes. Exosomes and miRNAs form a network that, both individually and synergistically, participates in the pathogenesis of PD among several other diseases (Mirzaei et al., 2017; Li et al., 2018; Saeedi Borujeni et al., 2018; Pourhanifeh et al., 2019).

Of note, certain miRNAs target PD-related genes. McMillan et al. (2017) observed that miR-7 can combine with the 3′-untranslated region (UTR) of SNCA mRNA to inhibit its transcription, and loss of miR-7 leads to α-syn aggregation and dopaminergic neuronal loss in the brains of PD patients (McMillan et al., 2017). Chen et al. (2017) found significantly upregulated expression of miR-4639-5p in PD patients, which negatively regulated the post-transcription level of the PD-associated gene DJ-1, eventually causing severe oxidative stress and neuronal death (Chen et al., 2017).

Jiang Y. et al. (2019) reported that exosomal microRNA-137 (miR-137) is upregulated and plays a vital role in the induction of oxidative stress of neurons in PD. miR-137 was found to directly target oxidation resistance 1 (OXR1) and negatively regulate its expression, thus inducing oxidative stress in PD (Jiang Y. et al., 2019). In a manganese-induced PD cell model, the levels of 12 miRNAs were significantly increased in EVs, including miR-210-5p, miR-128-1-5p, miR-505-5p, miR-325-5p, miR-16-5p, miR-1306-5p, miR-669b-5p, miR-125b-5p, miR-450b-3p, miR-24-2-5p, miR-6516-3p, and miR-1291. These miRNAs were shown to regulate key pathways in the pathogenesis of PD, including protein aggregation, autophagy, and inflammation (Harischandra et al., 2018). Toll-like receptor (TLR) is a type of innate immune receptor that when activated can cause release of inflammatory cytokines. Winkler et al. (2014) reported that exosomes can transport miRNA let-7 to activate TLR7 in neuronal cells and consequently lead to neurodegenerative diseases (Winkler et al., 2014).

Although the role of exosomes in the pathogenesis of PD has been confirmed, it is still necessary to explore the molecular mechanisms which control and regulate exosome biogenesis, secretion, and communication with recipient cells both *in vivo* and *in vitro*. Additional research concerning cell-to-cell transfer and propagation of α-syn, inflammatory mediators, and microRNAs between neurons and neuroglia cells, can broaden our understanding of the mechanisms of PD occurrence and progression, and allow for the development of new strategies for the diagnosis and treatment for PD and other neurodegenerative diseases.

## Role of Exosomes in the Diagnosis of PD

Nowadays, PD is mainly diagnosed by the appearance of noticeable clinical motor symptoms (Postuma et al., 2015). However, prior to the occurrence of motor symptoms, some non-motor symptoms are evident (Chaudhuri et al., 2006). There is currently no useful diagnosis for early stage PD. The development of a method to diagnose PD early would be an important breakthrough. Previous analysis of disease-related constituents of EVs (including exosomes) isolated from the blood or cerebrospinal fluid (CSF) of PD patients suggests that EVs can be efficient biomarkers of PD (Vella et al., 2016). The potential biomarkers in EVs of PD are summarized in Table 2 (ROC: Receiver Operating Characteristic Curve; AUV: Area Under the Curve).

**TABLE 2:** Potential biomarkers in EVs (including exosomes) of PD.

**EVs (including exosomes) sources**	**Potential biomarkers**	**Findings**	**ROC analysis**	**References**
CSF	α-syn	Lower in PD patients		Stuendl et al., 2016
	miR-153, miR-409-3p, miR-10a-5p, and let-7g-3p	Prominently increased in PD patients	AUC = 0.780; AUC = 0.970; AUC = 0.900	Gui et al., 2015
	miR-1 and miR-19b-3p	Prominently decreased in PD patients	AUC = 0.920; AUC = 0.705	Gui et al., 2015
Plasma	CNS-derived EV α-syn	Significantly higher in PD patients, and related to the severity	AUC = 0.654, sensitivity = 70.1%, specificity = 52.9%	Shi et al., 2014
	CNS-derived EV tau	Higher in PD patients compared with AD patients	AUC = 0.607, sensitivity = 57.8%, specificity = 65.1%	Shi et al., 2016
	CNS-derived EV DJ-1 and EV DJ-1/total DJ-1 ratio	Significantly higher in PD patients	AUC = 0.703, sensitivity = 79.5%, specificity = 57.5%; AUC = 0.724, sensitivity = 59.0%, specificity = 82.5%	Zhao et al., 2018
	Clusterin, apolipoprotein A1, complement C1r subcomponent	Significantly lower in HY stage II and PD III patients, apolipoprotein A1 is related to PD’s severity		Kitamura et al., 2018
	miR-331-5p	Prominently increased in PD patients	AUC = 0.849	Yao et al., 2018
	miR-505	Prominently decreased in PD patients	AUC = 0.898	Yao et al., 2018
Serum	Afamin, apolipoprotein D and J, pigmented epithelium-derived factor	Significantly higher in PD patients		Jiang R. et al., 2019
	Complement C1q, Immunoglobulin Lambda Variable 1-33 (IGLV1-33) Cluster -33	Significantly lower in PD patients		Jiang R. et al., 2019
	miR-24 and miR-195	Prominently increased in PD patients	AUC = 0.908, sensitivity = 81.7%, specificity = 85.0%; AUC = 0.697, sensitivity = 82.6%, specificity = 55%	Cao et al., 2017
	miR-19b	Prominently decreased in PD patients	AUC = 0.753, sensitivity = 68.8%, specificity = 77.5%	Cao et al., 2017
Saliva	phosphorylated α-syn	Significantly higher in PD patients		Rani et al., 2019
	α-syn oligomers and α-syn oligomers/total α-syn ratio	Higher in PD patients	AUC = 0.941, sensitivity = 92%, specificity = 86%; AUC = 0.772, sensitivity = 81%, specificity = 71%	Cao et al., 2019
Urine	DJ-1	Significantly higher in men in PD patients and increased in an age-dependent manner		Ho et al., 2014
	SerP-1292 LRRK2/total LRRK2 ratio	Predicted LRRK2 mutation status, higher in PD patients with LRRK2 mutation	AUC = 1.00, sensitivity = 100%, selectivity = 100%; AUC = 0.844, sensitivity = 100%, selectivity = 62.5%	Fraser et al., 2016a
	SerP-1292 LRRK2	Higher in men than women and increased in idiopathic PD patients, related to the severity of cognitive impairment		Fraser et al., 2016b

α-syn aggregation in LBs is a hallmark of PD pathology (Braak et al., 2003a). Although α-syn can be separated from blood and CSF, it cannot be usefully as a significant biomarker of PD due to its low abundance. In the CSF of PD patients, α-syn has consistently been shown to be present at a lower level compared to healthy controls. Stuendl et al. (2016) reported that levels of EV α-syn are also lower in the CSF of PD patients, which is consistent with the low levels of total α-syn in CSF (Stuendl et al., 2016). However, the use of plasma and serum α-syn as a biomarker has proved to be ineffective and inconsistent, because peripheral blood cells can also produce α-syn. Shi et al. (2014) reported that α-syn can easily be transported from the CSF to blood, and that some α-syn was packaged into EVs expressing neural cell adhesion molecule L1 (L1CAM), which is the central nervous system (CNS) specific (Shi et al., 2014). Moreover, they were able to demonstrate that levels of EV α-syn in plasma derived from the CNS are significantly higher in PD patients and the levels are related to the degree of the disease (Shi et al., 2014). In view of this, the authors suggest that CNS-derived EV α-syn in plasma can be used as a biomarker for PD with high specificity and sensitivity (Shi et al., 2014). Recently, Cao et al. (2019) found that the absolute levels of α-syn oligomers and the α-syn oligomers/total α-syn ratio are both higher in salivary EVs isolated from PD patients compared with healthy controls (Cao et al., 2019). Rani et al. (2019) were able to show that CNS-derived salivary EVs increased and that the levels of phosphorylated α-syn in these salivary EVs are significantly higher in PD patients than in healthy controls (Rani et al., 2019). Although Tau is mainly thought to be important in AD, Shi et al. (2016) reported that tau in CNS-derived EVs isolated from human plasma was significantly higher in PD patients compared with AD patients (Shi et al., 2016).

Kitamura et al. (2018) isolated EVs from the plasma of PD patients at Hoehn and Yahr (HY) stage II and III to identify candidate biomarkers for PD progression. They found that levels of clusterin, apolipoprotein A1, and complement C1r subcomponent were prominently decreased in PD patients at HY stage II and III compared with healthy controls. In particular, in PD patients at HY stage III, the levels of apolipoprotein A1 were substantially decreased compared with PD patients at HY stage II. Therefore, these three EV proteins may be candidate biomarkers for PD, it should be noted that apolipoprotein A1 levels are also relevant to the progression of PD (Kitamura et al., 2018). Similarly, Jiang R. et al. (2019) isolated EV proteins from the serum of PD patients, and observed that the expression of afamin, apolipoprotein D and J, and pigmented epithelium-derived factor, were prominently increased, whereas the levels of complement C1q and protein Immunoglobulin Lambda Variable 1-33 (IGLV1-33) Cluster -33 were decreased in PD patients (Jiang R. et al., 2019).

Mutations in DJ-1 and LRRK2 have been related to familial and sporadic PD. Zhao et al. (2018) reported that the levels of DJ-1 in EVs derived from CNS, and the ratio of EV DJ-1 to total DJ-1 derived from CNS, were substantially higher in the plasma of PD patients compared to healthy controls (Zhao et al., 2018). Ho et al. (2014) found that the levels of DJ-1 and LRRK2 in urine EVs in Korean PD patients are dependent on gender. Thus, DJ-1 levels were prominently higher in male PD patients and the levels increased in an age-dependent manner. However, the sample size in their study was small (Ho et al., 2014). Fraser et al. (2016a) examined whether the levels of auto-phosphorylated Ser(P)-1292 LRRK2 in urine EVs could predict LRRK2 mutation carriers (LRRK2+) and non-carriers (LRRK2-) with or without PD. The results revealed that these levels could predict LRRK2 mutation status using the elevated ratio of Ser(P)-1292 LRRK2 to total LRRK2 in urine EVs. Furthermore, patients with PD demonstrated a higher ratio than those without PD among carriers with the LRRK2 mutation (Fraser et al., 2016a). Overall, Fraser et al. (2016a) measured the levels of auto-phosphorylated Ser(P)-1292 LRRK2 in urine EVs in 79 idiopathic PD patients and 79 healthy controls. They found that the Ser(P)-1292 LRRK2 levels were higher in men compared to women and that they increased in idiopathic PD patients in contrast to healthy controls (Fraser et al., 2016a). Ser(P)-1292 LRRK2 levels were also shown to be related to the severity of cognitive impairment (Fraser et al., 2016b). Therefore, Ser(P)-1292 LRRK2 may be used as a biomarker for both familial and idiopathic PD.

In addition to proteins, various RNA species are present in EVs. Gui et al. (2015) isolated EV miRNAs from CSF, and revealed that 16 miRNAs were higher and 11 miRNAs were lower in PD patients in contrast to healthy controls. Among those, miR-153, miR-409-3p, miR-10a-5p, and let-7g-3p were prominently increased in CSF EVs from PD patients, while miR-1 and miR-19b-3p were prominently decreased (Gui et al., 2015). Cao et al. (2017) collected serum samples and isolated EV miRNAs from serum. They found that miR-24 and miR-195 were prominently upregulated, whereas miR-19b was prominently downregulated in serum EVs isolated from PD patients, as compared with healthy controls (Cao et al., 2017). Yao et al. (2018) found that the expression of circulating EV miR-331-5p was higher in the plasma of PD patients, while the expression of circulating EV miR-505 was lower, as compared with healthy controls (Yao et al., 2018).

The above studies suggest that EVs may be useful tools for the diagnosis of PD. However, large-scale clinical trials should be conducted to verify the specificity and sensitivity. Moreover, new techniques to isolate higher numbers of pure exosomes while excluding contaminants are required to improve the diagnostic value of exosomes.

## Role of Exosomes in the Treatment of PD

Currently, treatments for PD do not cure the disease, although many drugs can relieve the motor symptoms. However, as PD progresses, these drugs can produce adverse effects (Müller, 2012). Therefore, there is an urgent need for the discovery of new drugs or methods to treat PD.

**FIGURE 4:**
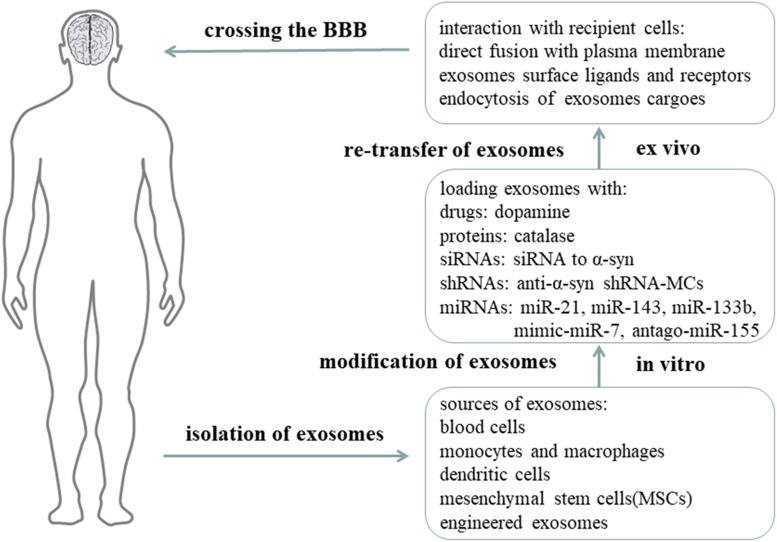
Exosomes as nano-delivery vehicles for PD treatment. It is proposed that exosomes can be used as nano-delivery vehicles for therapeutic drugs, proteins, siRNAs, shRNAs, and miRNAs. Exosomes are obtained from natural human cells or artificially synthesized, and then modified with therapeutic components *in vitro*, and finally re-injected into circulation. Exosomes can cross the BBB and reach the targeted cells to release their functional cargoes for therapeutic purposes (Aryani and Denecke, 2016).

Most of the drugs trialed for CNS diseases failed during clinical trials because that they cannot cross the blood-brain barrier(BBB) (Pardridge, 2012). However, exosomes can cross the BBB as natural nano-scaled vesicles, and they can be used as drug-delivery vehicles (Zhuang et al., 2011; Lai and Breakefield, 2012) (Figure 4). Qu et al. (2018) isolated exosomes from human blood, and loaded exosomes with a saturated solution of dopamine. In *in vivo* and *in vitro* experiments, the authors showed that blood exosomes could cross the BBB and deliver dopamine into the brain via an interaction between transferrin and transferrin receptor. The exosomes loaded with dopamine had a better therapeutic effect in the PD mice model and showed less toxicity than free dopamine by intravenously systemic administration (Qu et al., 2018). Haney et al. (2015) developed a new exosome delivery system loaded with catalase, which is a potent antioxidant, using monocytes and macrophages. These exosomes were taken up by neurons, and the catalase released could ameliorate neural inflammation and increase neural survival in *in vivo* or *in vitro* PD models (Haney et al., 2015). In addition, Kojima et al. (2018) reported a set of EXOsomal transfer into cells (EXOtic) devices that produces designer exosomes in engineered mammalian cells to deliver therapeutic catalase mRNA to the brain (Kojima et al., 2018).

Moreover, exosomes carrying specific exogenous small interfering RNAs (siRNAs) can be therapeutic for PD. In S129D α-syn transgenic mice, systemic injection of modified exosomes loaded with siRNA to α-syn can reduce the amount of α-syn mRNA transcription and protein translation (Cooper et al., 2014). Due to the short-term efficacy of siRNA, shRNA minicircles (shRNA-MCs) were designed by Izco et al. (2019). RVG-exosomes were used to deliver anti-α-syn shRNA-MCs to the PD mouse model induced by preformed α-syn fibrils. They found this treatment reduced the aggregation of α-syn, decreased the death of dopaminergic neurons, and ameliorated the clinical symptoms (Izco et al., 2019).

Exosomes derived from mesenchymal stem cells (MSCs) have been considered as an effective tool for treatment, and there use has been shown to be beneficial in different pathological conditions, including osteoarthritis (Mianehsaz et al., 2019), multiple sclerosis (Li et al., 2019), and PD. MSC-derived exosomes were discovered to rescue dopaminergic neurons in 6-OHDA mouse models of PD, providing a potential treatment for PD (Vilaca-Faria et al., 2019). MSC-derived exosomes can also carry beneficial miRNAs and interact with neuronal cells to reduce neuroinflammation and promote neurogenesis in PD animal models. miR-21 and miR-143 in MSC-derived exosomes are found to play a significant role in immune modulation and neuronal death. Moreover, as one of the miRNAs downregulated in PD, miR-133b in MSC-derived exosomes can be transmitted to neuronal cells to promote neurite outgrowth. In addition, by modifying MSC-derived exosomes with mimic-miR-7, it is possible to inhibit α-syn aggregation and suppress NLRP3 inflammasome activation in SNpc and striatum, thereby ameliorating the neuroinflammation response in PD. Modification with antago-miR-155 can also reduce microglia cell activation and neuroinflammation, and may be therapeutic for PD. Considering the above results, transfer of genetic materials such as miRNAs within MSC-derived exosomes, is indeed beneficial to PD animal models. Therefore, understanding how the miRNAs from MSC-derived exosomes interact with the cells and molecules in PD is of great importance.

Mounting evidence has been proved that exosomes separated from various types of cells can be modified to target specific neurons and specific regions of the brain and can be therapeutic for PD and many other neurodegenerative diseases (Tomlinson et al., 2015). Although the advantages of exosomes for therapy are apparent, some limitations exist. First, we cannot obtain pure exosomes using current technology, and so it is important for us to construct a minimum exosome delivery system to contain the therapeutic molecules and little else. Second, the adverse effects of using different sources of exosomes should be examined. Finally, we need to sort out the best cellular source for exosomes (Sarko and McKinney, 2017).

## Role of Exosomes in the Prognosis of PD

Not only do exosomes participate in the pathogenesis, diagnosis, and treatment of PD, exosomes can also be used as biomarker outcomes to show treatment response in PD clinical trials. In a single-center Exenatide-PD trial, 60 idiopathic PD patients were randomly assigned to subcutaneous administration of 2 mg exenatide (*n* = 31) or placebo (*n* = 29) once weekly for 48 weeks, followed by 12-week drug withdrawal. Blood samples were collected at week 0, 24, 48, and 60, and then neuronal-derived EVs with L1CAM were selectively isolated for insulin signaling proteins quantification. Athauda et al. (2019) found that patients receiving exenatide had enhanced brain insulin signaling with increased phosphorylation of insulin receptor substrate 1 (IRS-1) at 48 and 60 weeks, as compared with placebo controls. Moreover, the expression of downstream pathway substrates was increased, such as total phosphoinositide 3-kinase-protein kinase B (Akt) and phosphorylated mechanistic target of rapamycin (mTOR). In addition, they found that changes in levels of EV biomarkers including IRS-1 p-S616, t-mTOR, and p-mTOR S2448 were correlated with changes in Movement Disorders Society Unified Parkinson’s Disease Rating Scale (MDS-UPDRS) Part 3 scores at 48 weeks. At 60 weeks, the changes in MDS-UPDRS Part 3 scores were closely associated with changes in t-mTOR. These findings suggest that exenatide can improve the MDS-UPDRS Part 3 scores, and scores were significantly related to changes in these biomarkers in EVs (Athauda et al., 2019).

This research showed that changes in EV biomarkers were significantly associated with clinical improvements in an Exenatide-PD trial, and that EV biomarkers could potentially be used to evaluate treatment response and prognosis. Furthermore, neuronal-derived EVs could be a novel tool to assess target engagement for drugs in clinical trials in PD and other CNS diseases.

## Conclusion

Our in-depth analysis of exosomes reveals that these subcellular components participate in the onset, propagation and progression of PD, by spreading the harmful molecules, such as misfolded α-syn and inflammatory mediators. Isolation and identification of EV cargoes have been used to discover novel biomarkers for the diagnosis of PD. In addition, their ability to cross the BBB and the low immunogenic activity make exosomes ideal drug-delivery systems for the treatment of PD. Recent research suggests that neuronal-derived EVs isolated from peripheral blood can be used as biomarkers to assess prognosis and elucidate drug targets in clinical trials.

However, further investigations to elaborate the molecular mechanisms of exosomes in PD pathophysiology are warranted. What’s the direct mechanism for sorting α-syn into exosomes? In view of low levels of α-syn in exosomes, how many exosomes are required to propagate and cause pathology in *in vivo* or *in vitro* PD models? Are exosomes involved in the core pathogenesis of PD onset and progression, or are they released only as a result of PD pathophysiology? Most importantly, more accurate and standardized purification methods should be developed to isolate purer exosomes for diagnostic, therapeutic, and prognostic purposes. In 2011, the International Society of EVs was launched to tackle these challenges. We foretell that future research will shed light on these intriguing questions.

## Author Contributions

JF conceived, designed, and revised this review. HY was responsible for the original manuscript writing. JA and LW searched and collected the related references. FL, ZB, and YC drew the figures. TS completed the tables in this review.

## Conflict of Interest

The authors declare that the research was conducted in the absence of any commercial or financial relationships that could be construed as a potential conflict of interest.
